# Horse-disease on the Kankakee River

**Published:** 1897-02

**Authors:** 


SEVERAL large stock-raisers along the Kankakee River have suffered heavy losses during the past two weeks by valuable horses dying with an unknown disease. Up to date over one hundred have died. Many more are affected with the disease, which is unknown as yet to veterinarians. As soon as the disease attacks the animals their blood turns to water, and they soon die.

